# Swedish quality registry for caries and periodontal diseases (SKaPa): validation of data on dental caries in 6- and 12-year-old children

**DOI:** 10.1186/s12903-021-01705-x

**Published:** 2021-07-24

**Authors:** Tita Mensah, Sofia Tranæus, Andreas Cederlund, Aron Naimi-Akbar, Gunilla Klingberg

**Affiliations:** 1 Department of Pediatric Dentistry, Faculty of Odontology, Malmö University, 205 06 Malmö, Sweden; 2 The Clinic of Paediatric Dentistry, Region Värmland, Hagagatan 4-6, 652 20 Karlstad, Sweden; 3 Swedish Agency for Health Technology Assessment and Assessment of Social Services (SBU), P.O. Box 6183, 102 33 Stockholm, Sweden; 4 Health Technology Assessment Odontology (HTA-O), Faculty of Odontology, Malmö University, 205 06 Malmö, Sweden; 5 Department of Dental Medicine, Karolinska Institute, 171 77 Stockholm, Sweden; 6 Eastman Dental Institute, Stockholm County Council, Dalagatan 11, 102 31 Stockholm, Sweden

**Keywords:** Data accuracy, Registries, Child, Dental caries, Validation study, Diagnosis, Sensitivity and specificity

## Abstract

**Background:**

The Swedish Quality Registry for caries and periodontal disease (SKaPa) automatically collects data on caries and periodontitis from patients’ electronic dental records. Provided the data entries are reliable and accurate, the registry has potential value as a data source for registry-based research. The aim of this study was to evaluate the reliability and accuracy of the SKaPa registry information on dental caries in 6- and 12-year-old children.

**Method:**

This diagnostic accuracy study compared dental caries data registered at an examination with dental health status registered in the patient’s electronic dental records, and with corresponding data retrieved from the SKaPa registry. Clinical examinations of 170 6- and 12-year-old children were undertaken by one of the researchers in conjunction with the children’s regular annual dental examinations where the number of teeth were registered, and dental caries was diagnosed using ICDAS II. Teeth with fillings were defined as filled and were added to the ICDAS II score and subsequently dft/DFT was calculated for each individual. Cohen’s Kappa, the intraclass correlation coefficient (ICC), and sensitivity and specificity were calculated to test the agreement of the ‘decayed and filled teeth’ in deciduous and permanent teeth (dft/DFT) from the three sources.

**Results:**

Cohen’s Kappa of the dft/DFT-values was calculated to 0.79 between the researcher and the patient record, to 0.95 between patient dental record and SKaPa, and to 0.76 between the researcher and SKaPa. Intraclass correlation coefficient (ICC) was calculated to 0.96 between the researcher and the patient journal, to 0.99 between the patient dental record vs. SKaPa, and to 0.95 between the researcher and SKaPa.

**Conclusion:**

The SKaPa registry information demonstrated satisfactory reliability and accuracy on dental caries in 6- and 12-year-old children and is a reliable source for registry-based research.

*Trial registration *The study was registered in Clinical Trials (www.ClinicalTrials.gov, NCT03039010)

## Background

Oral and dental health are important components of general health, well-being, and quality of life [1]. In children and adolescents, dental caries is the most common chronic disease, often causing pain and requiring invasive interventions. In general, the dental health of Swedish children and adolescents is good. The Swedish National Board of Health and Welfare has compiled data on manifest caries at certain ages (3, 6, 12 and 19 years) annually since 1985. According to the last report for the year 2019, 72% of 6-year-olds and 67% of 12-year-olds in Sweden were caries-free [2]**.** A contributing factor is the free dental care for children and adolescents 0–19 years of age and the organization of dental services whereby the county councils have overall responsibility for delivery of dental care to children and young people. Approximately 95% of Swedish children and adolescents undergo regular dental examinations. The dental care is delivered by the Public Dental Service or by contracted private practitioners [3].

While most children are relatively healthy, some groups of children remain at higher risk for caries, with strong links to overall health and socio-economic factors [4]. One approach to the complex task of exploring such relationships is through registry-based studies. Official national registries are important tools for monitoring dental health in populations and also for registry-based research, in which data from different registries can be merged, to analyze relationships and to understand the interaction between dental health and different social factors, medical diagnoses and general health.

Sweden has a long history of national registries and registry-based research [5], made possible through the system of unique personal identity numbers, consisting of a twelve-digit-PIN, maintained by the National Tax Board for all individuals resident in Sweden since 1947. This identity number makes it possible to link data from different registries to a specific individual [6]. The more than one hundred national quality registries in Sweden are incorporated into clinical workflows and include individualized data about different medical interventions, procedures, and outcomes. They are thereby important tools not only for improving quality in the health sector but are also open for research [7].

As all dental records in Sweden are electronic and use the personal identity number as well as a national coding system to record diagnoses and treatment items [8], data on oral care are readily accessible for research. The Swedish Quality Registry for caries and periodontal disease (SKaPa) is a national registry started in 2008, initially involving Public Dental Services (PDS) at county level, but gradually extending nationwide to now include all PDS in Sweden and more and more private practitioners [9]. It currently includes data on about 90% of the child population (0–19 years of age) [10] and is the only dental registry in Sweden which includes children.

SKaPa is solely based on automatic retrieval of information from electronic dental records about all dental appointments (examinations, health status and treatments) that is subsequently delivered into the registry. This is different from most other registries where data are entered manually. All software systems for electronic dental records in Sweden can communicate with SKaPa. The registry stores information about dental clinics, dental health professionals and patients. Information at a patient level includes the personal identity number, sex, age, dental status on tooth level regarding diagnoses and treatments, risk assessments for caries and periodontitis, and dental care provided.

Although the SKaPa registry started the year 2008, information about validation of the registry has not been published. A prerequisite for using a register such as SKaPa for research is that one can trust the data extracted from the registry, therefore a validation of the quality of the data is of great importance. This validation study is the first systematic evaluation of the quality and validity of this type of dental registry with automatic retrieval of information from electronic dental records. As SKaPa covers such a large proportion of the population, and extensive datasets are available, the registry is potentially a valuable source of data for researchers, provided the validity is high. Knowledge from the study will be valuable for future studies where the SKaPa registry is used for research.

The main research question in this study was therefore: How reliable and accurate is SKaPa registry information on dental caries in children and adolescents?

The following hypothesis was tested: Comparison of clinical registrations with those in the electronic dental records, and with data retrieved from the SKaPa registry differ minimally regarding number of decayed and filled teeth (dft in the primary dentition and DFT in the permanent dentition) in 6 and 12-year-old children.

## Methods

After verbal and written information, the legal guardian signed an informed consent form. All children received age-appropriate information and consented to participation. The research was conducted ethically in accordance with the World Medical Association Declaration of Helsinki. The Regional Ethics Review Board in Uppsala, Sweden (#2016/051) approved the study. The study was also registered in Clinical Trials (www.ClinicalTrials.gov, NCT03039010).

### Study design

**Fig. 1:**
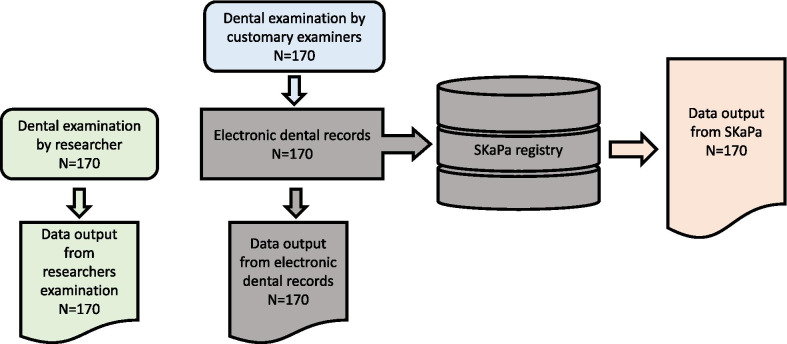
Schematic description of data collection. Every child was examined twice, independently by the researcher and the child’s customary examiner. The customary examiners entered the dental status in the electronic dental record. Comparisons were made between the researcher’s registrations, the data entered in the electronic dental records and the data retrieved from the SKaPa registry

In this diagnostic accuracy study the children were first examined by their customary examiners, who entered the dental status into the electronic dental record (which in this case uses the software *Carita*, Swedish Care®) followed by clinical examinations conducted by one of the authors (TM), a specialist in pediatric dentistry. Comparisons regarding dental caries were made between the researcher’s registrations, the data entered in the electronic dental records by the customary examiner and the data retrieved from the SKaPa registry. Thus, the study follows the entire chain of data: data from clinical dental examinations, dental health registrations in the electronic dental record and information on dental health retrieved from the SKaPa registry (Fig. 1).

### Setting and study population

This study was carried out in the County of Värmland, Sweden. The background child and adolescent population was about 55,000 with approximately 2,700 children in each age group (0–19 years old) at the time of data collection. The sampling procedure was undertaken in consultation with a biostatistician specialized in epidemiology, in order to make sure even distribution with respect to both socio-economic status and dental caries status, to ensure inclusion of children with expected higher prevalence of caries as well as relatively healthy children. The sample size was determined by using boot-strap resampling simulation based on a fictive sample size of 100,000 individuals. The simulation aimed to estimate a sample size that was enough in size given the initial proportion of 20 percent with caries and 80 healthy individuals. The resampled sample size increased for each iteration with 1 individual, where the true proportion of caries/healthy individual increased for each iteration. The cut-off was arbitrary based on the limitation of the number of possible participants in the study. The simulation showed that if the assumption of the proportion 20/80 for individuals with caries/healthy respectively is true, the sample of 75 is reasonable. The number of samples individual reflect a margin of error on the assumed proportion less than 10%. On this basis a final minimal study population was determined, of at least 75 children in each age category. Of the county’s 25 dental clinics 12 were chosen with enough children (> 10 per year group). These clinics included both rural and urban areas, child patients with different caries incidence, and reflected socioeconomic differences. Finally, 177 children were invited to participate: 83 6-year-olds, and 94 12-year-olds from twelve PDS clinics, with 11–17 children per clinic.

We also performed a post hoc sample size calculation to ensure a sufficient sample size. This showed that with a power of 0.90 and a significance level of 0.05 and to test the hypothesis that an assumed real ICC in the sample of 0.95 would differ significantly from a reference ICC of 0.90 we would need 83 subjects. To have at least that precision in each age group, our final sample size of 170 subjects is satisfactory.

### Training and calibration

Prior to the clinical examinations, the examiner (TM) underwent training and calibration in ICDAS II (International Caries Detection and Assessment system) scoring by e-learning (https://www.iccms-web.com) and radiographic registration of caries [11, 12]. ICDAS II is a standardized visual system for coding tooth decay. It provides options for coding six different stages of lesions (0 = sound, 1 = first visual change in enamel, 2 = distinct visual change in enamel, 3 = localized enamel breakdown, 4 = underlying dentin shadow, 5 = distinct cavity with visible dentin, and 6 = extensive cavity with visible dentin). The radiographic registrations were calibrated with another dentist from the research group, on 76 patients, a total of 152 radiographs. The calculated Kappa value was 0.79, with 95% confidence interval from 0.64 to 0.93. The strength of agreement was considered to be 'substantial'. Besides the e-learning, the examiner was also clinically trained and calibrated on ICDAS II. In a calibration exercise TM and another dentist from the research group independently examined ten children, all having teeth with as well as without carious lesions. For calibration as well as for TM’s clinical registration in the study each tooth was scored according to ICDAS II. A tooth with an ICDAS II score of 3–6 was defined as cavitated, i.e., a carious tooth. Teeth with fillings were defined as filled and were added to the ICDAS II score and subsequently dft/DFT was calculated for each individual. In the calibration a total agreement was reach on every tooth.

### Clinical examination

From December 2016 to March 2017, in conjunction with their annual dental check-ups, the participating children had an additional clinical examination by TM at their regular dental clinics. TM underwent an update and a new session in ICDAS scoring by e-learning prior to every visit to the different dental clinics. At the clinic, every child was first examined by the customary examiner at the clinic who decided, based on individual indications, whether radiographs were taken. This is the standard procedure and in accordance with legal requirements in Sweden. The children were examined in a normal dental clinic setting, seated in the dental chair using artificial light, a dental mirror and a probe. The teeth were dried with compressed air.

The customary examiner registered oral health status including caries in the electronic dental record. According to clinical guidelines manifest dental caries are defined as visible tooth substance loss without the characteristics of a developmental defect, or in pits and fissures when the point of the probe “caught” upon gentle pressure. Manifest radiographic caries is defined as lesion extending beyond the enamel-dentine junction. The child was then immediately re-examined by TM, registering the number of teeth and dental caries using the ICDAS II score and dft/DFT was calculated for each individual.

All registrations were blinded: the research examiner (TM) had no access to the registrations made earlier the same day by the customary examiner. TM used existing radiographs for diagnosis and no extra radiographs were taken for this study. Comparisons were made between the researcher’s (TM’s) registrations of dental health, the dental health status in the electronic dental records (customary examiner’s registrations), and the data retrieved from the SKaPa registry.

### Statistical analyses

To test the agreement in dft/DFT between the researcher’s dental examination, the data registered in the electronic dental record and the SKaPa registry, intraclass correlation coefficient (ICC) was calculated. Separate calculations were made of researcher examination/SKaPa, dental records/SKaPa and researcher examination/dental records. The calculations were stratified according to age, i.e. 6 and 12 years.

To test the accuracy of caries diagnostics at tooth level, calculations were made for sensitivity, specificity, predictive value of positive test (PV+), predictive value of negative test (PV−) with 95% confidence intervals. Also, Cohen’s Kappa was calculated for the agreement on tooth position level. Kappa was also used for calculation level of agreement of radiographic diagnosis in the calibrations prior to clinical examinations. All statistical analyses were performed using Stata 15 SE. P-values less than 0.05 were considered statistically significant.

## Results

Altogether 177 children (83 6-year-olds, 94 12-year-olds) from twelve PDS dental clinics were invited to undergo clinical examination in conjunction with their annual check-up. 171 children presented for examination. One declined to participate. All seven children not included in this study were reported to have been assessed as low caries risk at examination the year before. Finally, 170 children underwent examination: 84 boys (40 6-year-olds, 44 12-year-olds) and 86 girls (43 6-year-olds, 43 12-year-olds). Bitewing radiographs were taken by the customary examiner on 77 of the children (30 6-year-olds, 47 12-year-olds). Table 1 presents the mean value of the caries-index ‘decayed filled teeth’ (dft/DFT) reported by the researcher (TM), the electronic patient dental record and SKaPa, respectively.

**Table 1:** Decayed filled teeth (dft/ DFT-value) in 6- and 12-year-old children, reported by the researcher’s clinical examination, electronic patient dental record and SKaPa

	Total (n = 170)				6-year-olds (n = 83)				12-year-olds (n = 87)			
	Mean	Min–max	SD	Skewness	Mean	Min–max	SD	Skewness	Mean	Min–max	SD	Skewness
Researcher	0.68	0–8	1.48	2.82	0.94	0–8	1.93	2.11	0.43	0–4	0.77	2.16
Patient dental record	0.72	0–7	1.44	2.39	0.98	0–7	1.87	1.79	0.47	0–3	0.79	1.66
SKaPa	0.72	0–7	1.48	2.44	0.95	0–7	1.91	1.88	0.49	0–4	0.86	1.94

Agreement for dft/DFT-values on tooth position level for the three data categories (the researcher’s clinical examination, the electronic patient dental records and the SKaPa registry) was calculated using Cohens Kappa and revealed generally very good correlations (Table 2). Also, the intraclass correlation coefficient (ICC), which reflects the degree of agreement was calculated and revealed very good correlations (Table 3). Calculations of sensitivity and specificity also showed very good agreement (Table 4).

**Table 2:** Agreement for reported dft/DFT-value on tooth level between the researcher, the patient dental records and the SKaPa-registry (Cohen’s Kappa)

	Kappa (SE)	Agreement (%)
Researcher/patient dental record	0.91 (0.02)	99.4
Patient dental record/SKaPa	0.93 (0.02)	99.5
Researcher/SKaPa	0.87 (0.02)	99.2

**Table 3:** Intraclass Correlation (ICC) of reported dft/DFT in 6- and 12-year-old children between the researcher, electronic patient dental record and SKaPa

	Total (n = 170)		6-year-olds (n = 83)		12-year-olds (n = 87)	
	ICC	95% CI	ICC	95% CI	ICC	95% CI
Researcher/patient dental record	0.96	0.94–0.97	0.96	0.94–0.98	0.92	0.89–0.95
Patient dental record/SKaPa	0.99	0.98–0.99	0.99	0.98–0.99	0.97	0.95–0.98
Researcher/SKaPa	0.95	0.93–0.96	0.96	0.93–0.97	0.90	0.85–0.93

**Table 4:** Accuracy of caries diagnostics. Comparisons between researcher, patient dental record and SKaPa registry

	Sensitivity	95% CI	Specificity	95% CI	PV +	95% CI	PV –	95% CI
Researcher/patient dental record	0.93	0.87–0.97	1.00	0.99–1.00	0.90	0.83–0.94	1.00	1.00–1.00
Patient dental record/SKaPa	0.97	0.93–0.99	1.00	0.99–1.00	0.90	0.84–0.94	1.00	1.00–1.00
Researcher/SKaPa	0.93	0.87–0.97	0.99	0.99–1.00	0.83	0.76–0.89	1.00	1.00–1.00

Values for sensitivity, specificity, predictive value of positive test (PV+), predictive value of negative test (PV−) and 95% confidence intervals

## Discussion

The aim of this study was to disclose the level of agreement (regarding dft/DFT) between the researcher’s clinical dental examination, dental health status registered in the electronic dental record, and data retrieved from the SKaPa registry. The intraclass correlation coefficient (ICC) has previously been categorized as values less than 0.5 are indicating poor, between 0.5 and 0.75 as moderate, between 0.75 and 0.9 as good, and greater than 0.90 as excellent reliability [13].

Kappa has been categorized that values between 0.00–0.20 are indicating slight, 0.21–0.4 fair, 0.41–0.60 as moderate, 0.61–0.8 as substantial and 0.81–1.00 as almost perfect strength of agreement [14]. Our calculations of Cohen’s Kappa of the dft/DFT-value was calculated to 0.79 between the researcher and the patient record, to 0.95 between patient dental record and SKaPa, and to 0.76 between the researcher and SKaPa, indicating “substantial” to “almost perfect” agreement. The intraclass correlation coefficient (ICC) was calculated to 0.96 between the researcher and the patient journal, to 0.99 between the patient dental record vs. SKaPa, and to 0.95 between the researcher and SKaPa indicating “excellent” reliability. The results confirm the reliability and accuracy of the data in the SKaPa registry. The clinical status for dft/DFT in 6 and 12-year-old children differs minimally from data entered into the SKaPa registry, and from that retrieved from the registry.

SKaPa is a national quality registry in dentistry solely based on automatic retrieval and delivery of data from electronic patient dental records. The information is transferred via secure connections to the SKaPa registry or ‘data warehouse’ on a daily basis, which is an SQL (Structured Query Language) relational database [10]. There are other dental databases in the Scandinavian countries such as KOSTRA (Municipality-State-Reporting) data in Norway and the Danish Health Authority's Central Odontology Register (SCOR) system in Denmark [15]. These databases are built on manually collected reports of data on selected age groups. SKaPa currently includes data on about 90% of the whole child population in Sweden (0–19 years of age) [10].

Caries can be diagnosed clinically by visual inspection or by probing, supported by radiographic evidence, and more recently by adjunctive methods such as fiberoptic transillumination and laser fluorescence [16]. A combination of visual-tactile and radiographic examination is more reliable than either method used separately, but to date there is inadequate scientific support for other adjunctive diagnostic tools [17]. Intra-examiner reliability is usually higher than inter-examiner reliability when diagnosing dental caries [18].

In general, accuracy in excluding the presence of caries is greater than in confirming its presence [19]. Bitewing radiographs were taken on 77 of the children in this study. Radiographs should only be taken on individual indications, according to the standard procedure and the legal regulations in Sweden. As radiographs were unavailable for 93 of the children, there could be a risk that some caries lesions were undetected. Further, in all analyses, the lowest agreement was between the researcher and the SKaPa registry. This is what would be expected as is it the comparison with the most potential sources of error, with the differences between two examiners as well as the potential error in the data transfer.

The outcome measures in this study were the number of teeth with caries, expressed as dft and DFT in the two age groups, respectively. Originally it was intended to use the outcome measure deft/DMFT (including e = extracted, M = missing due to caries) but as there was a problem to retrieve e/M from the registry we assessed this variable to be unreliable and it was not included in this study.

It is important to consider possible shortcomings of using SKaPa registry data for this kind of study. The registry shows calendar year status, i.e. from 1st January to 31st December, so that when a restoration or an extraction is undertaken during the year the status registered in SKaPa changes. Therefore, the diagnostics made during the examinations did not always conform with the data extracted from SKaPa, which represents the accumulative status in 31st of December. In this study there were a few individuals with differences in dft/DFT in the electronic patient dental record and the data extracted from SKaPa, which in turn affects Cohen’s Kappa, ICC and calculations of specificity and sensitivity. Review of the dental records of these patients disclosed that they had developed new caries lesions during the calendar year, after the examination date, and as SKaPa displays the dft/DFT values for the full calendar year, the data differed. Another limitation could be that only ten children were examined for calibration before the clinical examinations. The WHO recommends at least 25 children for epidemiological studies. Yet, these ten children were not caries free and there was a perfect agreement on caries scoring.

Generally, the dental health of Swedish children is good. This can be a shortcoming in this kind of study, as it includes many children with good dental health. We have tried to accommodate this by distributing both socio-economic status and occurrence of dental caries through both urban and rural parts of Värmland.

Population-based dental registries make it possible to monitor dental health changes in a population. They are also important tools in health care research, for studying risk factors and evaluating interventions. As SKaPa holds information about diagnoses and treatment it is possible to use the registry for different types of research and in large groups of patient populations, e.g. longitudinal follow-ups of treatment, comparisons of oral health in different subgroups, exploring dental health in relation to general health issues by linking with other registries etc. Further, the registry is also available for international research collaborations, why its properties are of interest also outside of Sweden. However, quality registry-based research is possible only if registrations in patient dental records are complete and are transferred to the registries without error. There is always a risk of false variables in data records. Validation of registries is highly important, to ensure that the data are valid and reliable [20]. This was also the scope of the present study, and from that perspective it is both important and promising that this study shows very good correlation between the registered data and that retrieved from the SKaPa registry.

## Conclusions

The SKaPa registry information demonstrated satisfactory reliability and accuracy on dental caries in 6- and 12-year-old Swedish children and is a reliable source for registry-based research.

## Data Availability

We do not have ethical permission for unrestricted sharing of personal data outside our research group and national guidelines do not support unrestricted sharing of personal data. However, researchers have the possibility of applying for the datasets used and/or analyzed from the corresponding author on reasonable request. The data was retrieved from the SKaPa registry after ethical approval and application to the SKaPa registry.
